# Motor imagery EEG classification via wavelet-packet synthetic augmentation and entropy-based channel selection

**DOI:** 10.3389/fnins.2025.1689647

**Published:** 2025-11-10

**Authors:** Minmin Zheng, Zhengkang Qian, Tong Zhao

**Affiliations:** College of Intelligent Manufacturing, Putian University, Putian, China

**Keywords:** EEG, motor imagery, data augmentation, channel selection, transformer

## Abstract

**Introduction:**

Motor-imagery (MI) brain–computer interfaces often suffer from limited EEG datasets and redundant channels, hampering both accuracy and clinical usability. We address these bottlenecks by presenting a unified framework that simultaneously boosts classification performance, reduces the number of required sensors, and eliminates the need for extra recordings.

**Methods:**

A three-stage pipeline is proposed. (1) Wavelet-packet decomposition (WPD) partitions each MI class into low-variance “stable” and high-variance “variant” trials; sub-band swapping between matched pairs generates synthetic trials that preserve event-related desynchronization/synchronization signatures. (2) Channel selection uses wavelet-packet energy entropy (WPEE) to quantify both spectral-energy complexity and class-separability; the top-ranked leads are retained. (3) A lightweight multi-branch network extracts multi-scale temporal features through parallel dilated convolutions, refines spatial patterns via depth-wise convolutions, and feeds the fused spatiotemporal tensor to a Transformer encoder with multi-head self-attention; soft-voted fully-connected layers deliver robust class labels.

**Results:**

On BCI Competition IV 2a and PhysioNet MI datasets the proposed method achieves 86.81 and 86.64% mean accuracies, respectively, while removing 27% of sensors. These results outperform the same network trained on all 22 channels, and paired *t*-tests confirm significant improvements (*p* < 0.01).

**Discussion:**

Integrating WPD-based augmentation with WPEE-driven channel selection yields higher MI decoding accuracy with fewer channels and without extra recordings. The framework offers a computationally efficient, clinically viable paradigm for enhanced EEG classification in resource-constrained settings.

## 1 Introduction

The brain–computer interface (BCI) system establishes a communication pathway between the human brain and external devices, enabling the translation of users’ intentions into actions without relying on peripheral nerves or muscles (Xiong et al., 2021). Motor Imagery (MI), a prominent BCI paradigm, involves users actively imagining limb movements to activate corresponding brain regions and generate electroencephalogram (EEG) signals. Distinct motor imagery tasks produce differentiable EEG patterns, thereby facilitating the linkage between mental commands and external devices (Keutayeva et al., 2024). Accurate recognition and classification of MI-EEG signals are critical for advancing BCI systems toward practical daily applications. While EEG is convenient and inexpensive, practical MI decoding is hindered by three intertwined limitations. First, single-session recordings rarely exceed a few 100 trials, yielding a severely under-sampled condition. Second, the low signal-to-noise ratio of scalp measurements makes the relevant *α*/*β* components easily obscured by physiological or environmental artifacts. Third, high-density montages (64–128 electrodes) improve spatial resolution but prolong preparation and computation, conflicting with the portability and low-latency demands of mobile or home-use systems.

Motor-imagery (MI) EEG decoding has long been hampered by small sample sizes, high noise levels, and redundant channels. Data augmentation (DA) and channel selection (CS) are therefore routinely exploited to mitigate these problems. Early studies relied on hand-crafted features combined with digital filtering: the common spatial pattern (CSP) family—e.g., FBCSP (Chin et al., 2008), RCSP (Lotte and Guan, 2011), and TR-CSP (Samek et al., 2014)—decomposes the signal into multiple frequency bands or introduces regularization terms to derive spatial filters that simultaneously perform channel selection. To reduce filter leakage, CSP-based pipelines usually incorporate cross-validation on the training set together with symmetric subset selection, establishing the classical CS paradigm. With the advent of deep learning, both DA and CS have been integrated into end-to-end architectures. Representative DA techniques include CSGAN (Song et al., 2021), CovMix (Zoumpourlis and Patras, 2022) and OT-FMixup (Chen et al., 2022) augment motor-imagery EEG by interpolating or adversarially generating new samples along the time–frequency–spatial dimensions, enhancing cross-subject decoding without extra recordings. For CS, EEGNet (Lawhern et al., 2018) and ShallowConvNet (Schirrmeister et al., 2017) learn channel weights through the first 1-D convolutional layer, whereas TCNet-Fusion (Musallam et al., 2021) and M-FANet (Fu et al., 2024) attach attention gates; conformer (Song et al., 2023) and C2CM (Sakhavi et al., 2018) impose implicit pruning via grouped or sparse self-attention. Recent work further infuses neurophysiological priors into model training. Physics-informed Attention-TCN (Altaheri et al., 2022), for instance, constructs a frequency-domain loss term that aligns the network’s internal representations with the well-established *μ*/*β* rhythms of EEG, and jointly optimizes this loss with the data-driven objective to enhance interpretability. The Fine-Grained Spatial-Frequency-Time (SFT) (Liu et al., 2025) framework partitions 22 scalp channels into several region-of-interest groups guided by neuroanatomy, enabling multi-scale spatial feature extraction. Building on these advances, the present study aims to unify data augmentation and channel selection under a common neurophysiological prior.

To address these limitations, this paper proposes: a hybrid EEG data augmentation method integrating time-domain and frequency-domain techniques; a channel selection approach based on wavelet packet energy entropy difference for eliminating redundant channels; a multi-branch spatio-temporal convolutional Transformer network that effectively leverages the complementary advantages of multi-scale feature extraction across subjects, with final classification determined through a voting mechanism. First, we augment the EEG dataset by generating synthetic EEG data through our hybrid time-frequency augmentation method. Combining original and synthetic data effectively mitigates model overfitting. Subsequently, redundant channels are eliminated using our wavelet packet energy entropy difference-based selection method. For the model architecture, three parallel branches with distinct temporal convolutional scales extract local temporal features, followed by spatial convolution to capture spatial patterns. The resulting spatio-temporal features are processed by Transformer encoder modules, where multi-head self-attention mechanisms learn global temporal dependencies in EEG signals. Each branch produces independent outputs through separate fully connected layers, with final classification determined by majority voting. Experimental validation on public datasets confirms the effectiveness of our method in enhancing classification performance.

The principal contributions of this study are as follows:

1. EEG data augmentation method: A hybrid time-domain and frequency-domain EEG data augmentation approach designed to expand datasets and alleviate model overfitting.
2. EEG channel selection method: A wavelet packet energy entropy difference-based channel selection algorithm that eliminates redundant channels. This approach achieves comparable or superior classification performance to full-channel methods using fewer EEG channels. Evaluations of different channel group configurations demonstrate that symmetrically distributed channel groups yield optimal results.
3. Multi-branch spatio-temporal feature extraction architecture: A network design featuring three parallel temporal convolutional branches for multi-scale temporal feature extraction. Spatial convolution is subsequently applied to each branch. The spatio-temporal features are fed into Transformer networks where multi-head attention mechanisms dynamically assign weights. Final decisions are integrated via voting across fully connected layers.

## 2 Related work

Section 2 surveys the three computational pillars of modern MI-EEG decoding: data augmentation, channel selection, and deep representation learning. For each pillar we trace the evolution from heuristic or handcrafted solutions to recent learning-based strategies, highlight residual bottlenecks, and thereby motivate the integrated framework proposed in this work.

### 2.1 Data augmentation

Early EEG augmentation relies on geometric transforms—rotation, flipping, and electrode swapping—to enlarge datasets. Shovon et al. (2019) rotate and flip MI-EEG trials in a multi-input CNN, though these operations risk discarding fine temporal–spectral information. Zhang et al. (2019) first convert EEG to STFT spectrograms, then apply rotation and noise injection, enhancing geometric augmentation for image-based pipelines. Roy et al. (2019) swap left–right electrodes within the same class, yielding modest benefits. Roy et al. (2020) employ GANs for subject-specific synthesis, requiring heavy adversarial training. Qin et al. (2023) use Static-Var Generation (SVG) to model electrode displacements and spatial brain patterns, densifying samples near the original manifold; the impact of exact electrode positioning needs further study.

Decomposition–fusion strategies exploit deeper EEG structure. Yu and Stefan (2023) exchange time segments between same-class trials and swap matched frequency bands, achieving joint temporal–spectral augmentation. Xia et al. (2024) apply DCT, randomly recombine six selected frequency bands, and reconstruct plausible synthetic trials. EEG augmentation has thus progressed from simple geometric manipulations to sophisticated decomposition–fusion schemes respecting spatio-temporal–spectral properties. Motivated by these advances, we propose an integrated framework coupling wavelet-packet decomposition with geometric crop-and-reconstruction. Relevant motor-imagery nodes are exchanged in the frequency domain before signal reconstruction, preserving physiological validity while expanding the class-conditional distribution. Subsequent geometric cropping further blends these samples, yielding rich yet physiologically consistent augmentations.

### 2.2 Channel selection

Traditional EEG channel selection typically relies on neuroscientific brain parcellations for manual electrode selection over task-relevant regions. However, substantial inter-subject variability often renders such selections prone to redundant or irrelevant sensors (Blankertz et al., 2008). Modern approaches are broadly categorized as wrapper-based or filter-based strategies.

Wrapper methods optimize channel subsets using classifier performance as the objective, employing heuristic searches. Wei and Wang (2011) applied BMOPSO to motor-imagery EEG, reducing 22 channels to eight. Zhang et al. (2021) used Deep MOPSO with CNN accuracy, trimming to 10 channels with ~3% accuracy gain. Vadivelan and Sethuramalingam (2025) coupled MPJS with DB-EEGNet, compressing 64 channels to 12 and boosting accuracy from 79.3 to 85.6%. These methods yield tailored subsets but are computationally expensive and prone to overfitting.

Filter methods derive relevance scores directly from signals for pruning. Lotte and Guan (2011) ranked channels by energy contribution to CSP filters. Arvaneh et al. (2011) introduced sparse CSP with L₁ regularization, achieving 85% accuracy with only 8 of 22 channels. Filter methods are computationally efficient and resource-frugal.

Balancing both approaches and inspired by CSP, this study adopts a wavelet-packet energy-entropy-based filter. This method captures energy distribution and uncertainty with minimal computation, addressing CSP’s exclusive focus on second-order energy differences and neglect of phase/connectivity information.

### 2.3 Deep learning models

Influenced by deep learning, MI-EEG decoding now centers on CNNs, RNNs and Transformers, substantially lifting accuracy (Bicchi et al., 2021). Schirrmeister et al. (2017) introduced end-to-end spatio-temporal CNNs as the baseline. Sakhavi et al. (2015) employed FBCSP pre-processing followed by CNN classification, yet hand-crafted features remain a bottleneck. Amin et al. (2019) fused global and local representations via layer-wise weighted convolutions; Roy (2022) proposed a multi-scale CNN to harvest discriminative cues within separate frequency bands. Nevertheless, these CNN-centric models still struggle to capture long temporal dependencies.

Transformers, renowned for long-range modeling, are rapidly adopted. Adel et al. (2024) used self-attention across spatial–temporal dimensions, periodically updating channel weights and improving accuracy. Ma et al. (2022) coupled CNNs with Transformers: CNNs extract local patterns and Transformers capture global dependencies across spatial, spectral and temporal domains. Luo et al. (2023) presented a shallow mirrored Transformer with multi-head self-attention and left–right mirroring for data augmentation. Permana et al. (2023) designed a dual-level architecture in which a low-level Transformer extracts short-time features and a high-level Transformer attends to salient intervals via dynamic weighting.

We integrate multi-branch CNNs and Transformers: parallel temporal convolutions with multiple receptive fields capture multi-scale local dynamics, providing temporal priors for the Transformer. The Transformer then establishes global dependencies and adaptively weights features. This hybrid design mitigates the limited long-range modeling of pure CNNs and the local-detail oversight of pure Transformers, enabling more robust and accurate decoding under scarce data and high inter-subject variability.

In summary, augmentation has evolved from simple geometric transforms to decomposition–fusion schemes that respect EEG physiology; channel selection has moved from anatomical heuristics to data-driven ranking; and deep models have shifted from pure CNNs to CNN–Transformer hybrids that couple local spectral cues with long-range context. To the best of our knowledge, this study is the first to simultaneously expand the class-conditional manifold via wavelet-domain node exchange, compress the electrode array with wavelet-packet energy–entropy filtering (without classifier retraining), and feed the enriched yet concise data into a multi-branch CNN–Transformer for accurate, lightweight and robust MI decoding in small-sample, low-density scenarios.

## 3 Methods

**Figure 1:**
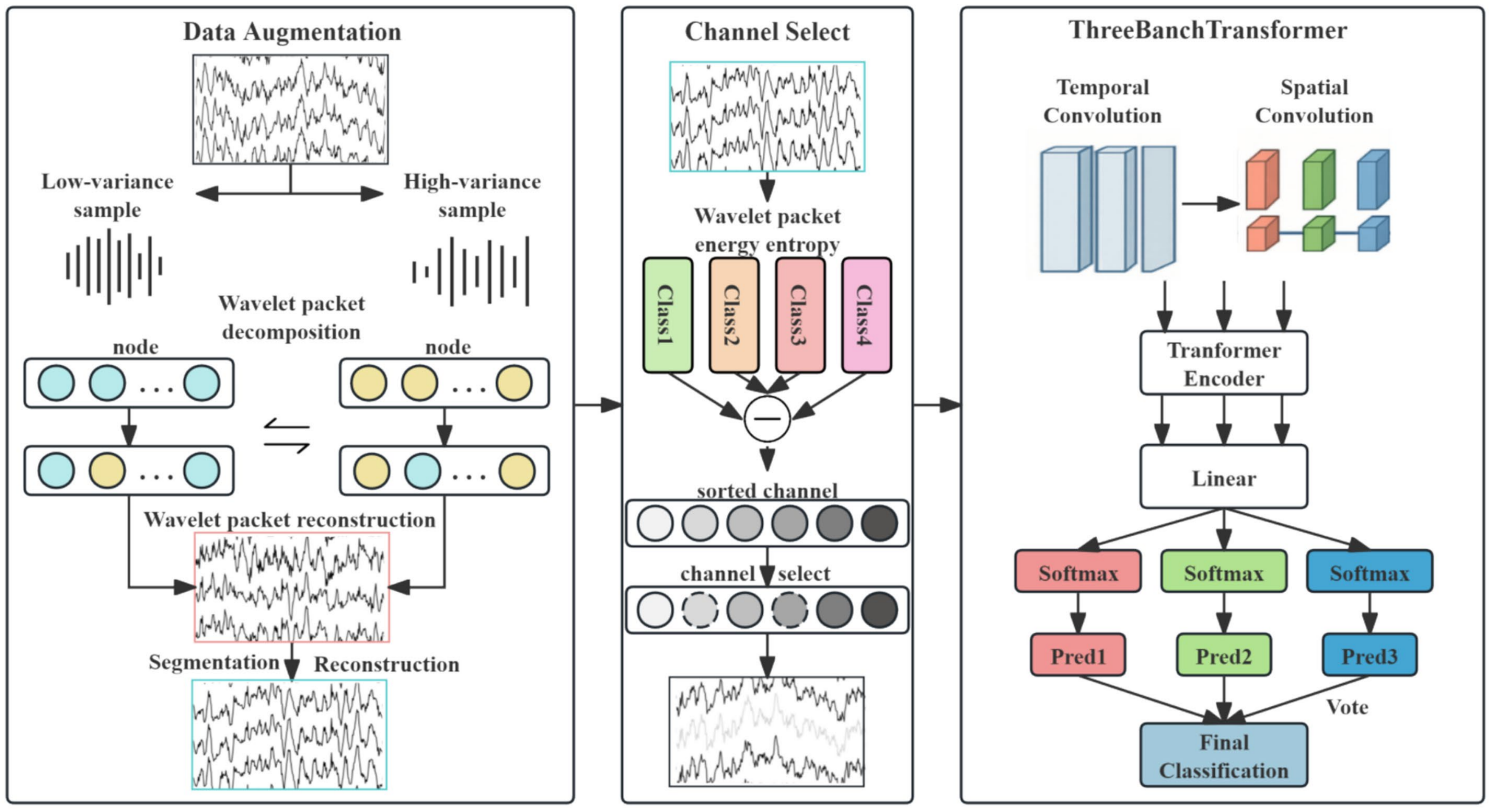
Flowchart of the DACS-TBTrans framework.

This study proposes a novel neural network architecture, as illustrated in Figure 1. The overall model architecture integrates four core components: data preprocessing and augmentation, channel selection, a three-branch spatio-temporal convolutional Transformer module, and a voting classification module.

### 3.1 Overview

In the data preprocessing and augmentation stage, a hybrid EEG augmentation approach combining geometric cropping-reconstruction with decomposition-transform techniques is employed. This method effectively expands the scale of the EEG dataset, thereby mitigating model overfitting. Subsequently, the channel selection module utilizes a wavelet packet energy entropy difference-based method to identify and eliminate redundant channels.

The processed data then enters the three-branch spatio-temporal convolutional Transformer module. Within each branch, temporal convolutions with distinct kernel sizes extract multi-scale temporal feature representations. Spatial convolution is subsequently applied along the channel dimension to the selected EEG channels to enhance discriminative spatial features. Batch normalization and average pooling operations are incorporated after convolution to improve model stability and emphasize salient feature characteristics.

The resulting multi-scale spatio-temporal features from each branch are independently encoded by separate Transformer encoders. Multi-head attention layers within these encoders capture long-term temporal dependencies inherent in the EEG signals. Following Transformer encoding, the features from each branch are processed by distinct fully connected layers. Branch-specific classification probabilities are then computed via Softmax activation functions.

Finally, the voting classification module determines the ultimate class prediction through majority voting based on the classification outputs from the three individual branches.

### 3.2 Preprocessing and data augmentation

The public dataset was pre-processed as follows. Continuous EEG signals were first high-pass filtered at 1 Hz (zero-phase FIR) to remove drift, then notch-filtered at 50 Hz to suppress power-line interference, and finally band-pass filtered between 4 and 40 Hz using a sixth-order Chebyshev Type-II filter to attenuate physiological artifacts such as ECG and EMG. To enlarge the data volume, each trial was segmented—via an overlapping sliding window—into two 4-s epochs (2–6 s and 3–7 s post-cue) that were treated as independent samples, effectively doubling the dataset. It should be noted that this filter-only pipeline follows the conformer baseline and cannot remove ocular, cardiac, or muscular artifacts whose spectra overlap with the *α*/*β* bands; consequently, within-subject results may be slightly inflated. More sophisticated artifact-reduction methods (e.g., ICA or ASR) will be explored in future work to further validate the findings.

**Figure 2:**
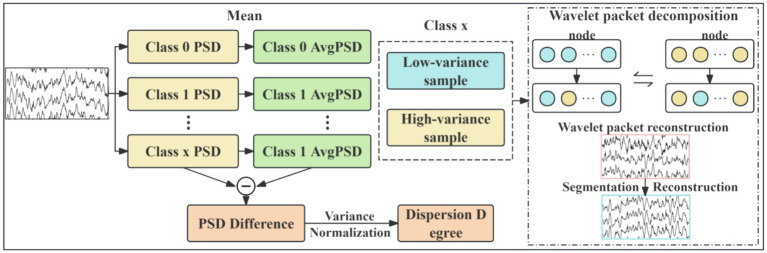
Flowchart of the data augmentation.

EEG data augmentation serves as an effective strategy for expanding datasets and enhancing model robustness. This study proposes a novel EEG data augmentation method based on power spectral density (PSD) analysis and time-frequency component exchange. It is critical to emphasize that this augmentation was applied exclusively to the training data in a strict manner to prevent any data leakage. The core approach involves, on the training set only, intra-class sample grouping, followed by the targeted exchange of specific frequency band components between low-variance samples (representing stable EEG patterns) and high-variance samples (representing variant EEG patterns). This process generates synthetic EEG signals possessing neurophysiological plausibility, which are then used to augment the training set. The held-out test set remains completely untouched and unaltered throughout this process, ensuring the validity of our evaluation. The overall workflow, illustrated in Figure 2, comprises the following steps:

1. Power spectral density calculation and intra-class grouping: compute PSD for each sample and categorize them into low-variance and high-variance groups within each class.
2. Wavelet packet decomposition for time-frequency representation: decompose the EEG signals using wavelet packet transform (WPT) to obtain their time-frequency representations.
3. Directed frequency band component exchange: swap specific frequency band components between paired low-variance and high-variance samples.
4. Wavelet packet reconstruction for synthetic sample generation: reconstruct the modified time-frequency components to generate new synthetic EEG samples.

For EEG data $X_{k}\in {\mathbb{R}}^{N_{k}\times C\times T}$ within each class k∈0,1,2,3 , the PSD is computed as:

$$P_{i}^{\left(k\right)}=\mathrm{PSD}(X_{i}),i=1,\ldots ,N_{k}$$ (1)

In Equation 1, $N_{k}$ represents the number of samples within the class *k*, *C* denotes the number of channels, *T* indicates the number of time points, and $P_{i}^{\left(k\right)}\in {\mathbb{R}}^{C\times F}$ is the PSD matrix of sample *i* (where *F* is the number of frequency points).

For each class, the mean PSD is computed as shown in Equation 2:

$${\bar{P}}^{\left(k\right)}=\frac{1}{N_{k}}\sum_{i=1}^{N_{k}}P_{i}^{\left(k\right)}$$ (2)

For each sample *i* and channel *c*, the normalized deviation is computed as shown in Equation 3:

$$\begin{array}{c} \Delta_{i,c}^{\left(k\right)}=P_{i,c}^{\left(k\right)}-{\bar{P}}^{\left(k\right)}\\ \Delta_{\mathrm{norm},i,c}^{\left(k\right)}=\frac{\Delta_{i,c}^{\left(k\right)}-\min(\Delta_{i,c}^{\left(k\right)})}{\max(\Delta_{i,c}^{\left(k\right)})-\min(\Delta_{i,c}^{\left(k\right)})} \end{array}$$ (3)

Where $P_{i,c}^{\left(k\right)}$ denotes the PSD corresponding to the *c* channel of the *i* sample, $\Delta_{i,c}^{\left(k\right)}$ represents the PSD deviation of the c channel in the *i* sample, and $\Delta_{\mathrm{norm},i,c}^{\left(k\right)}$ is the normalized deviation.

In Equation 4, the total variance metric is computed for each sample, and the dataset is partitioned into two groups:

$$\begin{array}{c} V_{i}^{\left(k\right)}=\sum_{c=1}^{C}\mathrm{Var}(\Delta_{\mathrm{norm},i,c}^{\left(k\right)})\\ G_{\mathrm{low}}^{\left(k\right)}=\{X_{i}^{\left(k\right)}|V_{i}^{\left(k\right)}\leq \text{median}(V^{\left(k\right)})\}\\ G_{\text{high}}^{\left(k\right)}=\{X_{i}^{\left(k\right)}|V_{i}^{\left(k\right)}>\text{median}(V^{\left(k\right)})\} \end{array}$$ (4)

Where $V_{i}^{\left(k\right)}$ denotes the total variance of the *i* sample in class *k*, samples are partitioned into two groups based on ascending total variance: the low-variance group $G_{\mathrm{low}}^{\left(k\right)}$ and high-variance group $G_{\text{high}}^{\left(k\right)}$ .

For each class of EEG signals, 5-level wavelet packet decomposition is performed using the db4 wavelet basis. Subsequently, directed frequency band exchange is implemented. Given that motor imagery EEG information is primarily concentrated in the *α* rhythm (8–13 Hz) and *β* rhythm (14–30 Hz) (Wu et al., 2025), sample pairs are formed by matching the sample with the lowest variance in $G_{\mathrm{low}}^{\left(k\right)}$ to the sample with the highest variance in $G_{\text{high}}^{\left(k\right)}$ using end-to-end correspondence. The time-frequency data of wavelet decomposition nodes corresponding to the *α* and *β* rhythm frequency bands are exchanged between each pair. Finally, wavelet packet reconstruction is applied to generate the frequency-augmented synthetic sample X′ .

For temporal domain augmentation, the geometric cropping-reconstruction method (Schirrmeister et al., 2017) is applied to both the frequency-augmented synthetic samples X′ and original EEG data **X**. Within each class, random cropping and splicing reconstruction are performed, generating augmented EEG data per class. These class-specific augmented data are then randomly shuffled and combined, yielding spatio-temporally augmented samples X″ . Finally, X″ is incorporated into the EEG dataset, producing the enhanced EEG data $\hat{X}$ .

### 3.3 Channel selection

EEG signals often contain redundant channels. Selecting channels that better represent the subject’s actual motor imagery information from the original set can significantly enhance model classification performance (Guo et al., 2025). This study proposes an EEG channel selection technique based on wavelet packet energy entropy analysis for channel screening. Crucially, channel selection is performed solely on the training data: the optimal channel subset is identified from the training set and then frozen for subsequent use on the test set, ensuring unbiased evaluation. As illustrated in Figure 1, the method quantifies each channel’s discriminative power in EEG signal classification by integrating energy distribution complexity with inter-class separability, enabling efficient feature selection. The procedure comprises four stages: wavelet packet decomposition and energy entropy computation, energy entropy standardization, inter-class divergence measurement, and comprehensive channel importance ranking.

First, a 5-level wavelet packet decomposition is applied to the EEG signals. In Equation 5, the energy of each channel is computed, followed by the calculation of energy entropy based on the energy proportion of each channel across decomposition nodes:

$$H_{i,c}=-\sum_{j=1}^{32}p_{i,c,j}\log_{2}p_{i,c,j}$$ (5)

Where *p_i,c,j_* denotes the energy proportion of channel *c* at decomposition node *j* for sample *i*, and *H_i_*, *c* represents the energy entropy of channel *c* for sample *i*.

In Equation 6, the energy entropy values undergo max–min normalization:

$${\tilde{H}}_{i,c}=\frac{H_{i,c}-\min_{m}H_{i,m}}{\max_{m}H_{i,m}-\min_{m}H_{i,m}}$$ (6)

Where ${\tilde{H}}_{i,c}$ denotes the normalized energy entropy, $H_{i,m}$ represent the maximum and minimum energy entropy values, respectively.

The normalized energy entropy is grouped by k∈0,1,2,3 class, with ${\tilde{H}}_{k}$ indicating the normalized energy entropy for class *k* data. Inter-class divergence is measured using the energy entropy values across four classes. Higher energy entropy values indicate more uniform energy distribution, signifying richer spectral information in that channel. We compute pairwise differences between class-specific channel energy entropy values, yielding six sets of channel energy entropy differences, which are then standardized:

The six standardized difference sets are sorted to obtain g∈0,1,2,3,4,5 descending-order channel sequence $D_{g}$ . Larger wavelet packet energy entropy differences indicate greater spectral divergence between corresponding channels of two classes. We posit that selecting the top *n* channels with the largest differences enhances discriminability of inter-class features, which benefits classification performance.

Based on the positions of the EEG electrodes, predefined pairs of symmetric electrodes, such as C3/C4, FC1/FC2, etc., are established. After calculating the wavelet packet entropy and obtaining the sequence of channels, the first *n* channels are selected (it is later verified that *n* = 12). Then, following the principle of electrode pairing, the process starts from the end of the channel sequence and works backward to match symmetrically until symmetry is achieved, thus completing the channel selection.

The channel indexes $D_{g}^{\prime }$ obtained through initial screening are comprehensively ranked by importance according to their spatial positions, yielding the final selected n channels $D_{g}^{\prime }$ .

### 3.4 Network architecture

As illustrated in Figure 1, the proposed method employs a multi-scale spatio-temporal feature extraction design. The input signals are processed in parallel through temporal convolutional layers in three branch networks. Each temporal convolution uses distinct filter sizes: (1 × 20), (1 × 40), and (1 × 60), respectively, with a uniform stride of (1 × 1). The outputs of these temporal layers are then fed into corresponding spatial convolutional layers, all utilizing kernel sizes of (*n* × 1) and stride (1 × 1) to extract spatial features.

Following multi-scale spatio-temporal convolution, each branch undergoes Batch Normalization and Exponential Linear Unit (ELU) activation to stabilize training and mitigate overfitting. To accommodate the varying scales of spatio-temporal features extracted by different branches, average pooling layers are applied with branch-specific configurations: Branch 1: Pool size (1 × 30), stride (1 × 25); Branch 2: Pool size (1 × 50), stride (1 × 20); and Branch 3: Pool size (1 × 70), stride (1 × 15). The processed features from all three branches are passed through separate fully connected layers to generate classification probabilities. Final class prediction is determined by majority voting. Experimental validation on public datasets confirms the effectiveness of this architecture in enhancing classification performance.

### 3.5 Performance metrics

To comprehensively evaluate the performance of the proposed method, datasets were partitioned into training and test sets. Models were trained on the training set and evaluated on the test set. For each subject, this procedure was performed independently, with results averaged across subjects. Three established metrics were employed: accuracy (Li et al., 2021), Kappa coefficient (Carletta, 1996), and F1-score (Li et al., 2019). Their computational formulations are given below:

$$\mathrm{Acc}=\frac{\mathrm{TP}+\mathrm{TN}}{\mathrm{TP}+\mathrm{TN}+\mathrm{FP}+\mathrm{FN}}$$ (7)

Accuracy (Acc) quantifies the proportion of correctly predicted samples relative to the total sample size. In Equation 7, TP denotes True Positives, TN denotes True Negatives, FP denotes False Positives, and FN denotes False Negatives. The value of Accuracy falls within the range [0, 1], where values closer to 1 correspond to superior model performance.

$$\kappa =\frac{P_{o}-P_{e}}{1-P_{e}}$$ (8)

The Kappa coefficient ( κ ) is computed from the confusion matrix to quantify the agreement between actual classifications and random chance classifications, thereby assessing model performance. In Equation 8 $P_{o}$ : Overall classification accuracy (observed agreement), calculated as the ratio of correctly classified samples to total samples. $P_{e}$ : Expected accuracy under random chance, derived from the product of marginal class distributions. The κ value ranges from −1 to 1, where values approaching 1 indicate increasingly reliable model performance beyond random chance.

$$F_{1}=\frac{2\mathrm{TP}}{2\mathrm{TP}+\mathrm{FP}+\mathrm{FN}}$$ (9)

In Equation 9, the F_1_ ranges between [0,1], with values approaching 1 indicating superior classification performance.

### 3.6 Experimental settings

The computational platform configuration comprised an Intel Core i9-11900k CPU and NVIDIA RTX A4000 GPU with 16GB VRAM. The software framework was implemented using PyTorch on Python 3.9 under Windows 11. Models were optimized using the Adam algorithm with batch sizes of 288 for Dataset 2a and 100 for Physionet MI, a learning rate of 0.0002, and cross-entropy loss. To prevent overfitting, training employed early-stop (patience = 50), weight-decay = 1 × 10^−5^, and a dropout rate of 0.5. The multi-head attention module utilized 10 heads with 6 layers, and training proceeded for 2000 epochs considering computational resources and model performance.

The BCI Competition IV 2a dataset comprises EEG signals from 9 subjects. Each subject performed four motor imagery (MI) tasks: left hand (Class 0), right hand (Class 1), tongue (Class 2), and feet (Class 3). Each session included 48 trials, with MI tasks equally distributed across classes. Six runs were conducted per subject, yielding 288 MI trials per subject. EEG data acquisition began at 2 s post-cue onset and continued until approximately 7 s, followed by a rest period. Given the standard 4-s analysis window (*T* = 1,000 sampling points at 250 Hz) and inter-subject variability, data segments from both 2–6 s and 3–7 s intervals were utilized, as both contain discriminative MI information. These intervals were merged to effectively double the dataset size.

The Physionet MI dataset (Goldberger et al., 2000) includes four MI tasks (left hand, right hand, fists, feet) recorded from 109 subjects. EEG data were acquired using 64 electrodes at 160 Hz. Each subject underwent baseline recordings (eyes open/closed) and completed three experimental sessions, each containing 14 trials. After excluding Subjects 38, 88, 89, 92, 100, and 104 due to inconsistent sampling rates or labeling errors, the 0–4 s post-visual-cue interval was extracted as input data.

## 4 Experimental and results

### 4.1 Results of the public datasets

#### 4.1.1 Results of the BCI competition IV dataset 2a dataset

The proposed DACS-TBTrans method was evaluated on two four-class motor imagery datasets: the BCI Competition IV Dataset 2a and the Physionet MI Dataset. Experimental results were compared against multiple state-of-the-art baseline methods, with statistical significance assessed using paired t-tests.

**Figure 3:**
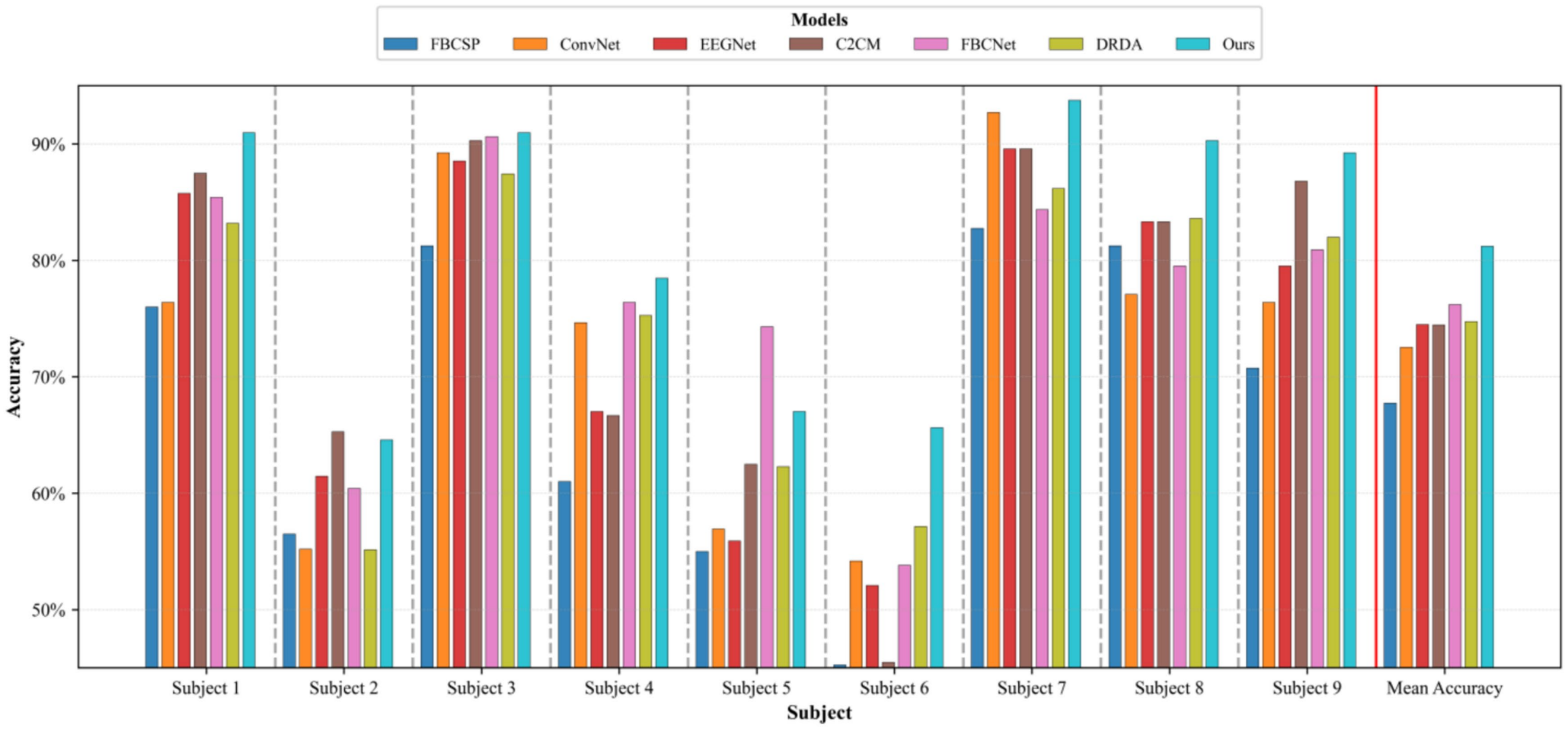
Comparative classification accuracy of proposed method vs. baselines on BCI competition IV 2a dataset.

The classification performance of the proposed method (DACS-TBTrans) on the BCI Competition IV 2a dataset is comprehensively presented in Table 1 and Figure 3. In this experiment, which used overlapping time windows to augment the dataset, the proposed method (DACS-TBTrans) achieved the highest classification accuracy among comparative algorithms for Subjects 1, 4, 6, 7, 8, and 9. It also demonstrated a significant improvement in average accuracy over other methods. This consistent outperformance across all subjects suggests that our approach effectively captures the nuances of EEG signal patterns associated with motor imagery tasks.

**Table 1:** Comparative classification accuracy (%) of proposed method vs. baselines on BCI competition IV 2a dataset.

Method	Sub1	Sub2	Sub3	Sub4	Sub5	Sub6	Sub7	Sub8	Sub9	*Avg*	κ	*P*
FBCSP (Chin et al., 2008)	76.00	56.50	81.25	61.00	55.00	45.25	82.75	81.25	70.75	67.75	0.5700	0.000011***
C2CM (Sakhavi et al., 2018)	87.50	65.28	90.28	66.67	62.50	45.49	89.58	83.33	79.51	74.46	0.6595	0.003101**
DRDA (Sakhavi et al., 2018)	83.19	55.14	87.43	75.28	62.29	57.15	86.18	83.61	82.00	74.74	0.6632	0.000047***
M-FANet (Fu et al., 2024)	86.81	**75.00**	91.67	73.61	76.39	61.46	85.76	75.69	87.17	79.28	0.7259	0.006200**
conformer (Song et al., 2023)	88.19	61.46	93.40	78.13	52.08	65.28	92.36	88.19	88.89	78.66	0.7155	0.023454*
TCNet fusion (Musallam et al., 2021)	90.74	70.67	**95.23**	76.75	**82.24**	68.83	94.22	88.92	85.98	83.73	0.7800	0.045601*
DACS-TBTrans	**91.67**	74.65	94.10	**88.54**	80.90	**70.83**	**97.57**	**93.06**	**89.93**	**86.81**	**0.8125**	**–**

Bold values indicate the best performance achieved for that metric.The symbols are denote statistical significance levels: *p <0.05. **p < 0.01, ***p < 0.001.

While models like FBCSP, C2CM, and M-FANet are capable of extracting features from EEG signals, they may not fully capture the long-term temporal dependencies that are crucial for accurate classification. Similarly, DRDA, though it enhances model robustness by incorporating data from other subjects, still lags behind our method in classification accuracy for every subject. Although TCNet Fusion achieved the highest accuracy in Subjects 3 and 5, our method attained the second highest. Given our method’s significant superiority in Subjects 1, 4, 6, 7, 8, and 9, TCNet Fusion’s overall accuracy remains lower than ours. This is evident from the high accuracy rates across multiple subjects, particularly in Subjects 4 and 7, where it achieved 93.06 and 97.57% accuracy, respectively.

The superior performance of the proposed method primarily stems from its frequency-domain-based data augmentation technique, which generates reliable synthetic EEG data, combined with its multi-scale temporal feature extraction approach. Figure 3 further substantiates this efficacy through clear comparative visualization.

To statistically validate the performance superiority demonstrated in Table 1, paired *t*-tests were conducted comparing the proposed method with baselines on the BCI Competition IV 2a dataset The results indicate that the proposed method consistently outperforms all baseline methods with statistically significant differences. Specifically, it shows extremely statistically significant improvements over FBCSP (*p* ≤ 0.001), and DRDA (*p* ≤ 0.001), highly statistically significant improvement over M-FANet (*p* ≤ 0.01), and C2CM (*p* ≤ 0.01), statistically significant improvements over conformer (*p* ≤ 0.05) and TCNet Fusion (*p* ≤ 0.05). These results strongly support the superiority of the proposed method, demonstrating that the enhancements in classification accuracy are not due to chance but are statistically validated.

**Figure 4:**
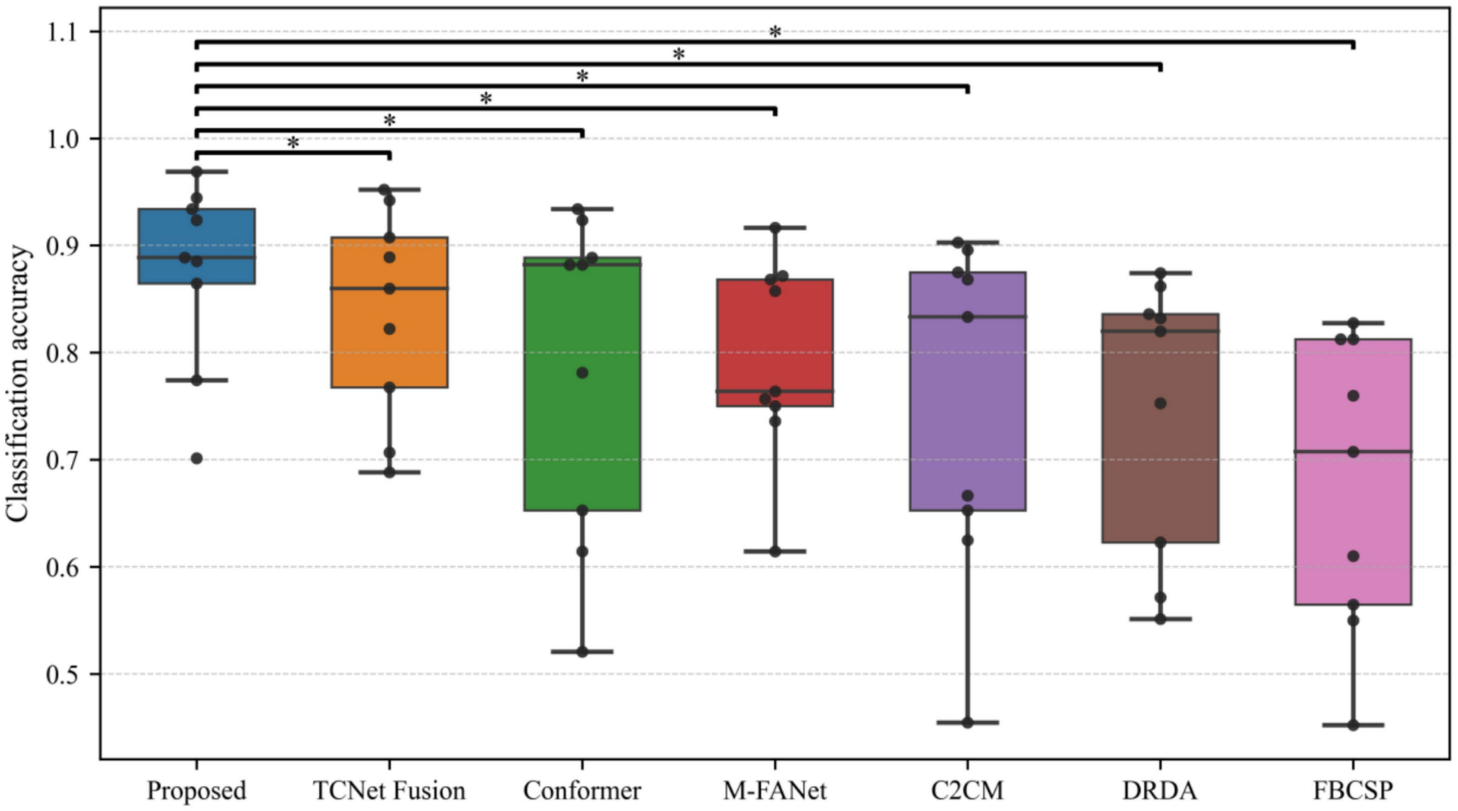
Model performance comparison with statistical significance.

**Figure 5:**
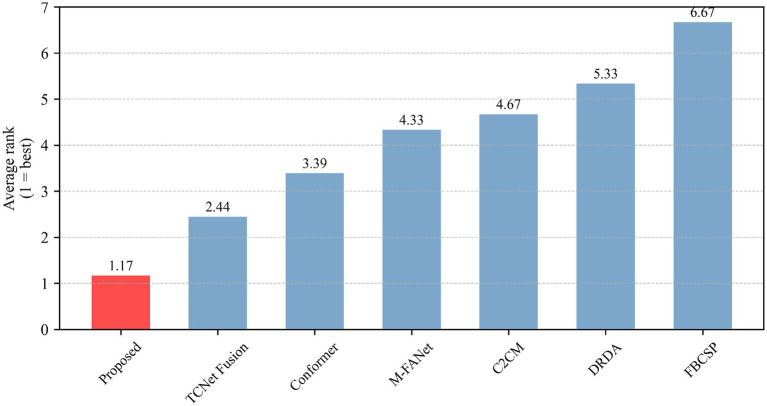
Model ranking based on Friedman test.

In motor-imagery (MI) EEG classification, the accuracy scores obtained by the same subjects under different models are inherently paired; however, small sample sizes, non-normal distributions, and heavy-tailed or outlier-prone differences frequently violate the assumptions (normality and homoscedasticity of the differences) required by the paired *t*-test, inflating Type I error or diminishing statistical power. A non-parametric procedure that relaxes distributional assumptions while controlling multiple-comparison error is therefore essential. The Wilcoxon signed-rank test demands only symmetry of the paired differences; it ranks the absolute discrepancies and restores the signs, fully exploiting the paired structure and remaining robust to outliers—properties especially advantageous for small-sample MI datasets. Holm–Bonferroni correction, a step-down family-wise error rate (FWER) control procedure, offers higher power than the classic Bonferroni method yet maintains strict control over the overall error rate without resampling. Accordingly, we performed pairwise comparisons between the proposed model and six baselines: for each pair, subject-wise accuracy differences were first computed and evaluated with a two-tailed Wilcoxon signed-rank test to obtain raw *p*-values; these *p*-values were then adjusted by Holm–Bonferroni correction, and significance was claimed at *α* = 0.05. The outcomes of this analysis are illustrated in Table 2, Figures 4, 5, where stars mark the pairs that remain significant after correction (*p* < 0.05), providing rigorous statistical evidence for the performance improvement achieved by the proposed MI classifier.

**Table 2:** Statistical results (Wilcoxon signed-rank test with Holm–Bonferroni correction).

Comparison	*p-raw*	*p-adj (Holm)*	CliffΔ	Effect
Proposed vs. FBCSP	0.0039	0.0234	0.8024	Large
Proposed vs. C2CM	0.0039	0.0234	0.5556	Large
Proposed vs. DRDA	0.0039	0.0234	0.7037	Large
Proposed vs. M-FANet	0.0039	0.0234	0.5802	Large
Proposed vs. conformer	0.0117	0.0234	0.3951	Medium
Proposed vs. TCNet fusion	0.0078	0.0234	0.2346	Small

All raw *p*-values were ≤ 0.001 (Wilcoxon signed-rank test). After Holm–Bonferroni correction, every comparison yielded an adjusted *p* < 0.001, so Table 2 reports identical values; the corresponding *p*-values obtained from paired *t*-tests are provided in Table 1.

Our model underwent the Wilcoxon signed-rank test, Holm–Bonferroni correction, and ranking based on the Friedman test. As can be seen from Table 2, all comparisons (between the proposed model and other models) have *p*-values less than 0.05 after applying the Holm–Bonferroni correction, indicating that these comparisons are statistically significant. That is, the proposed model shows a significant difference in classification accuracy compared to other models. The effect size indicates a large effect for comparisons between the proposed model and FBCSP, C2CM, DRDA, and M-FANet, suggesting that the proposed model performs significantly better in these comparisons. The comparison between the proposed model and conformer shows a medium effect size, indicating that although the proposed model performs better, the difference is not as significant as in the previous comparisons. The comparison between the proposed model and TCNet Fusion shows a small effect size, indicating a smaller performance difference between the two. After applying the Holm–Bonferroni correction, the *p*-values for all comparisons are slightly higher than the original *p*-values but still less than 0.05, demonstrating that the differences between the proposed model and other models remain significant even when controlling for multiple comparison errors. The identical adjusted *p*-values may be due to the original *p*-values of each comparison being very close, resulting in the same ranking and thus the same correction factor. It can be observed from the figures that our model exhibits stable classification performance across subjects, with low variability in the data. It performs the best in both classification accuracy and average ranking, and the differences compared to other models are statistically significant.

**Figure 6:**
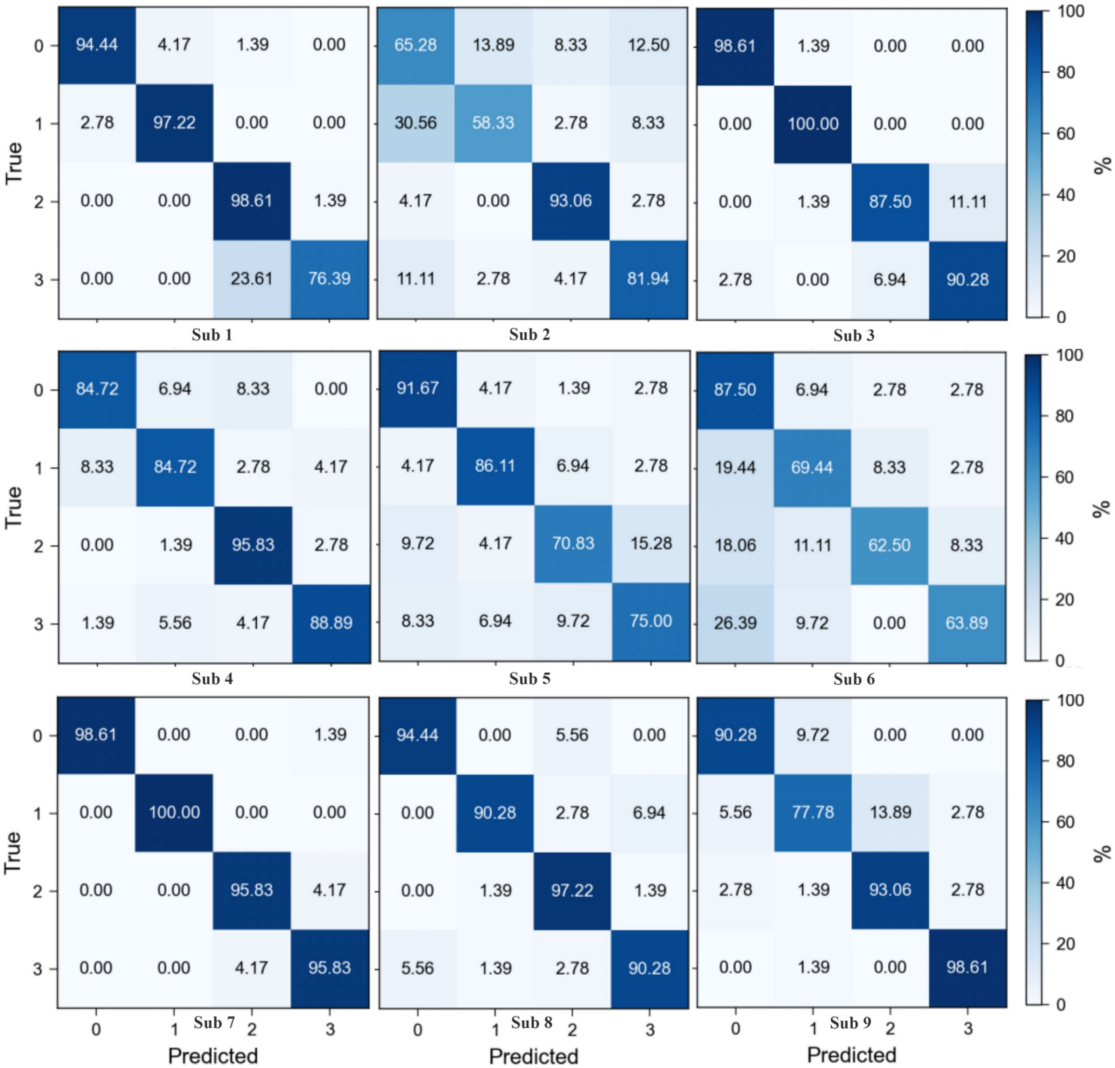
Confusion matrix of proposed method on BCI competition IV 2a dataset.

The confusion matrices for the Motor Imagery Dataset 2a (Figure 6) reveal a varied classification performance across subjects, highlighting the complexity of EEG signal decoding. Subjects 1, 3, 8, and 9 demonstrate superior classification accuracy, as evidenced by the high values along the diagonal of their respective matrices, indicating a robust discrimination capability of the proposed method in decoding EEG signals associated with motor imagery tasks. In contrast, Subjects 2, 4, 5, and 6 exhibit relatively poorer performance, with a notable increase in off-diagonal elements, suggesting a challenge in distinguishing between certain motor imagery classes. Notably, for Subject 2, Class 2 (tongue motor imagery) achieves the highest classification accuracy, aligning with previous findings that suggest a distinct neurophysiological signature for tongue motor imagery tasks. The observed misclassification between tongue (Class 3) and feet (Class 4) motor imagery tasks in Subjects 1, 3, 7, and 8 could be attributed to suboptimal channel selection, which potentially misses critical EEG features, thus warranting further investigation. The performance discrepancies across subjects underscore the necessity for subject-specific optimization strategies and highlight the importance of refining channel selection algorithms to enhance classification accuracy. Future work should focus on adapting the channel selection process to account for inter-subject variability in EEG signal characteristics, aiming to reduce misclassification rates and improve the overall robustness of EEG-based motor imagery decoding.

#### 4.1.2 Results of the Physionet MI dataset

**Figure 7:**
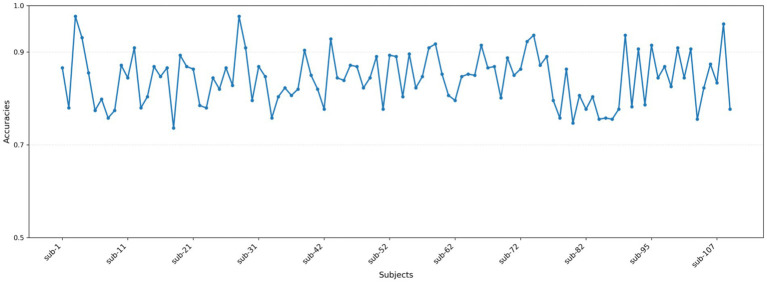
Classification accuracy of each subject in the Physionet MI dataset.

The classification outcomes of the proposed method on the Physionet MI dataset, as detailed in Table 2 and depicted in Figure 7, showcase a marked improvement over existing baseline methods. These results were derived from an analysis that excluded data from Subjects 38, 88, 89, 92, 100, and 104 due to inconsistencies in sampling rates or labeling errors, leaving a total of 103 valid subject datasets for evaluation. The proposed method not only demonstrates a significantly higher average classification accuracy but also achieves peak accuracy of 97.61% in individual subjects, culminating in an overall average accuracy of 86.64%. This performance surpasses that of other state-of-the-art methods such as FBCSP, DeepConvNet, ShallowConvNet, EEGNet, EEGNet fusion, and MI-EEGNe, as evidenced by the comparative metrics presented. The superior performance of the proposed method can be attributed to its advanced feature extraction capabilities and robust classification strategy, which effectively leverage the nuances within EEG signal patterns associated with motor imagery tasks. This analysis underscores the proposed method’s potential as a leading approach in EEG-based motor imagery classification, offering enhanced accuracy and reliability for brain–computer interface applications.

**Figure 8:**
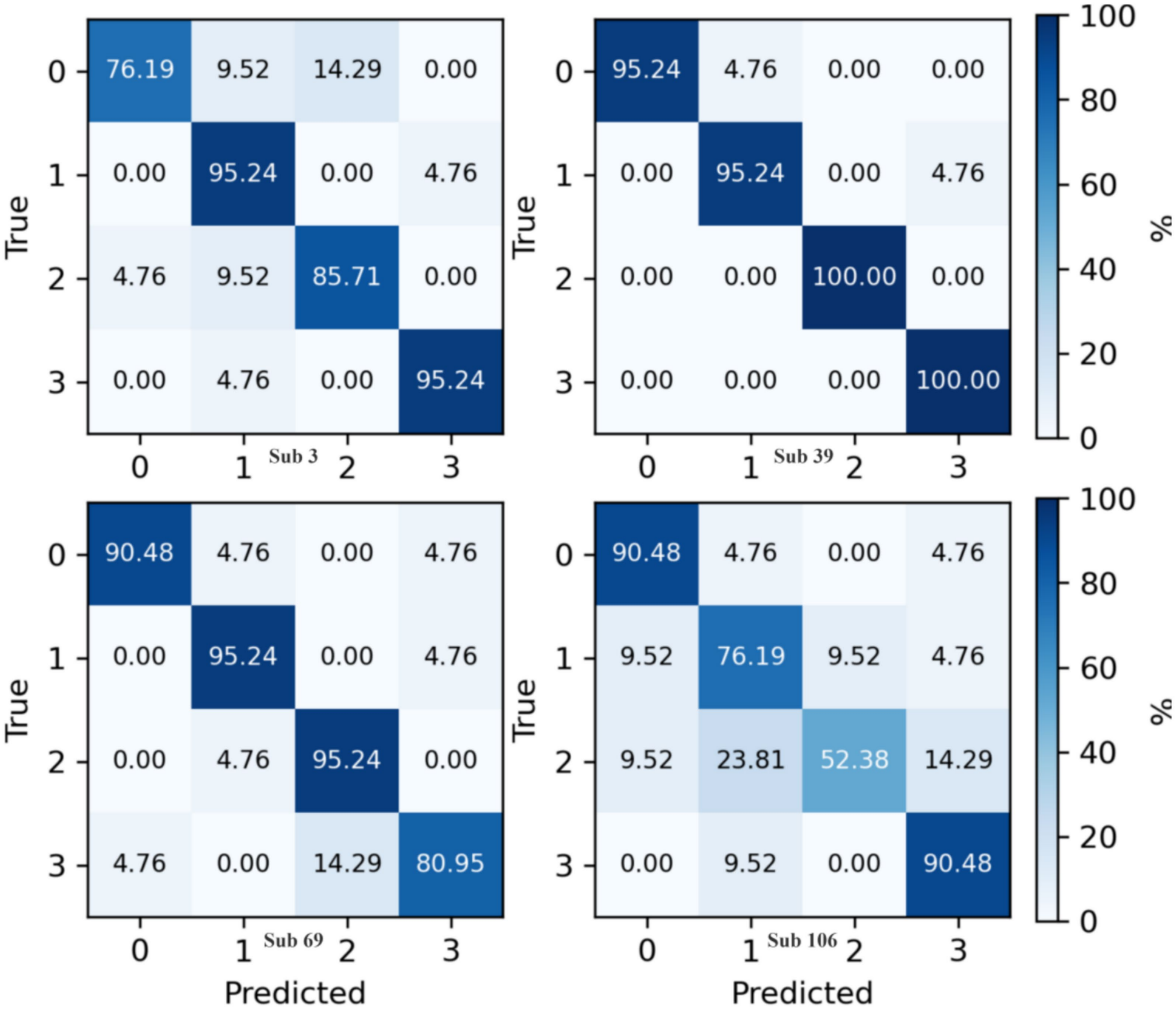
Confusion matrix of proposed method on Physionet MI dataset.

The confusion matrix for the proposed method on the Physionet MI dataset is presented in Figure 8. This matrix aggregates predictive outcomes from 10-fold cross-validation to generate the final classification results for each subject. Due to the extensive number of subjects, representative samples from Subjects 3, 39, 69, and 106 were selected for detailed analysis. Notably, Subject 106 exhibits significant intermixing between Class 1 and Class 2 data, corroborated by its relatively lower classification accuracy in Figure 5. This observation aligns with prior findings (Shuqfa et al., 2024), which document data recording anomalies and abnormally short trial durations for Subject 106. Consequently, exclusion of this subject’s data is methodologically justified.

### 4.2 Results of the ablation experiment

The methodology of our study is anchored by two essential components: sophisticated data augmentation strategies and selective channel elimination, which are pivotal for enhancing EEG signal classification. To evaluate the impact of these components, we executed a meticulous ablation study on the BCI Competition IV 2a dataset. This involved sequentially removing each component from our model and measuring the performance against a baseline multi-scale three-branch network model. The experimental findings are systematically captured in Figures 7, 8, along with detailed results in Table 3. These visualizations and data underscore the critical contribution of both data augmentation and channel selection to our model’s superior classification performance, thereby affirming their indispensable roles in optimizing EEG-based motor imagery classification.

**Table 3:** Comparative classification accuracy (%) of proposed method vs. baselines on Physionet MI dataset.

Method	*Avg*	F1-score
FBCSP (Chin et al., 2008)	30.63	0.30
DeepConvNet (Schirrmeister et al., 2017)	57.66	0.57
ShallowConvNet (Schirrmeister et al., 2017)	70.16	0.70
EEGNet (Lawhern et al., 2018)	37.53	0.36
EEGNet fusion (Zhao et al., 2021)	73.73	0.74
MI-EEGNet (Riyad et al., 2021)	85.71	0.86
DACS-TBTrans	86.64	0.86

**Figure 9:**
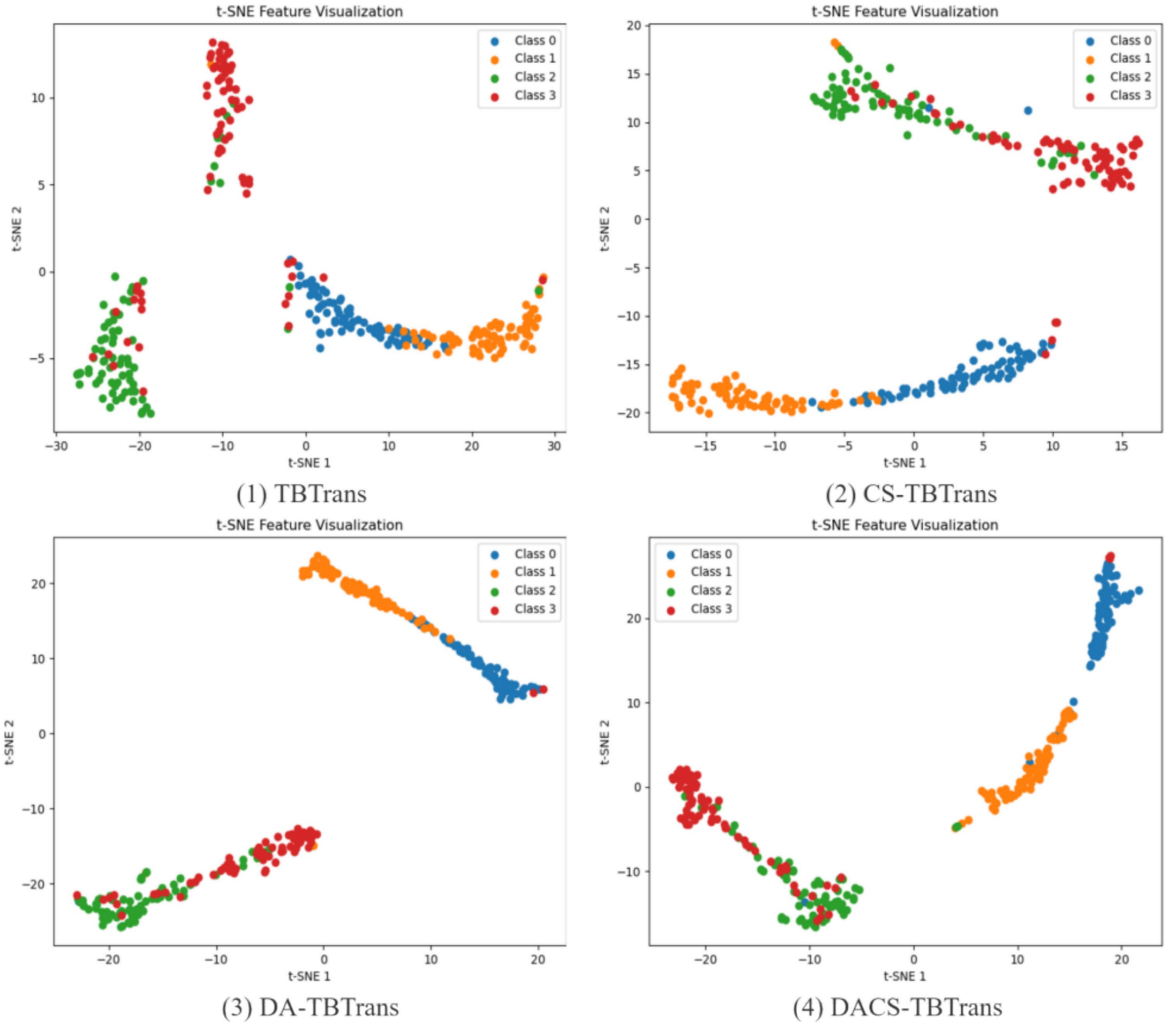
Feature visualization of proposed method for Subject 1 on BCI competition IV 2a dataset.

The analysis presented in Figure 9 illustrates the feature visualization for Subject 1 on the BCI Competition IV 2a dataset, highlighting the comparative effectiveness of the proposed DACS-TBTrans method against the baseline TBTrans model. It is evident that the standalone implementation of channel selection and data augmentation modules independently contribute to enhancing feature clustering across different classes. This improvement is characterized by a notable reduction in feature overlap when juxtaposed with the TBTrans model. The DACS-TBTrans approach exacerbates these positive effects by not only further concentrating class-specific features but also by achieving a successful isolation between Class 0 and Class 1. This strategic differentiation bolsters classification accuracy, although a considerable overlap between Classes 2 and 3 remains, pinpointing a specific area that necessitates further refinement and optimization.

**Figure 10:**
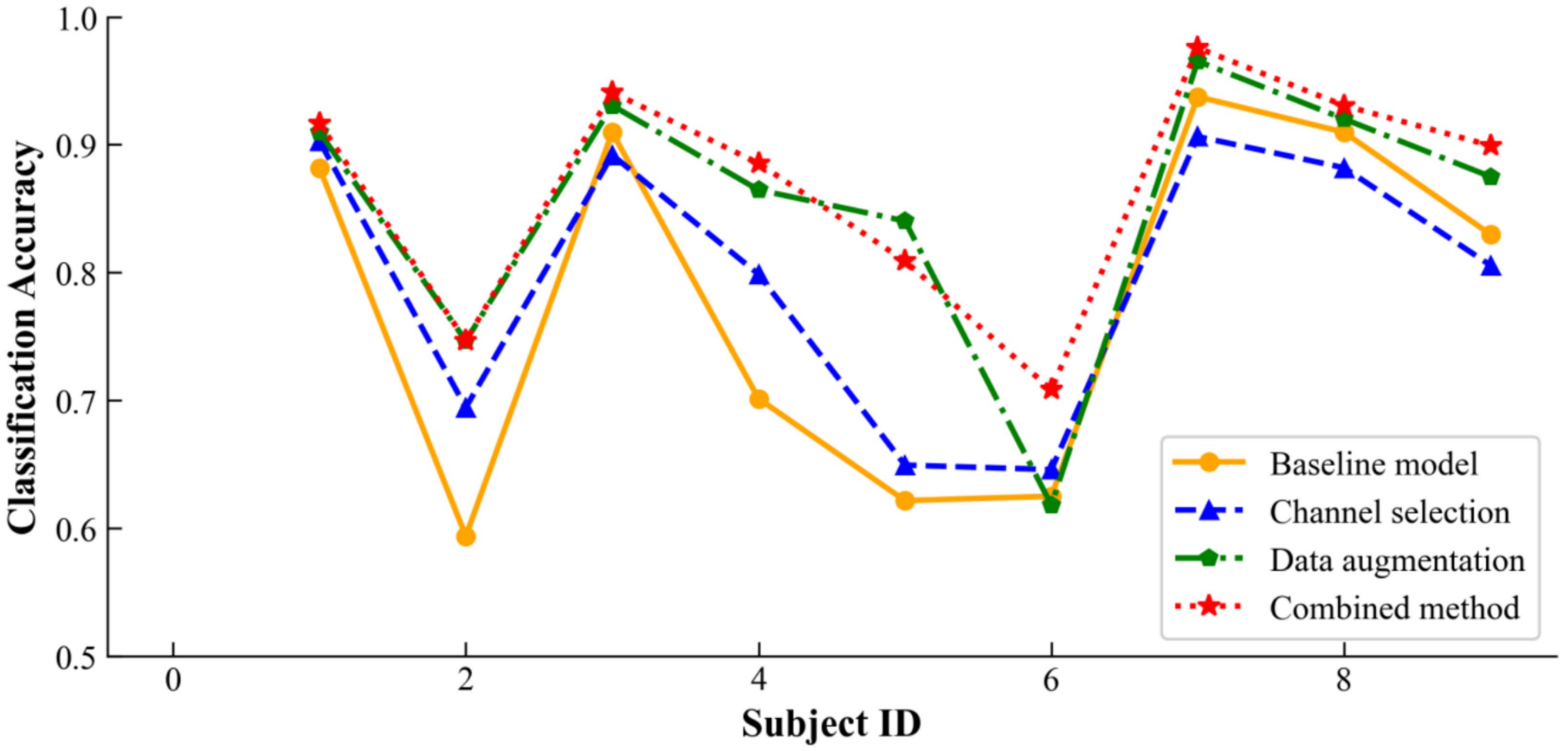
Ablation results of proposed method on BCI competition IV 2a dataset.

As a supplement, Figure 10 illustrates how incorporating channel selection or data augmentation influences classification accuracy across a broader cohort of subjects. The results show that channel selection is particularly beneficial for Subjects 2, 4, and 5, as it effectively removes redundant channels and thereby improves the signal-to-noise ratio. Similarly, data augmentation delivers significant advantages for Subjects 2, 4, 5, 7, and 9 by enhancing the dataset’s robustness against overfitting on EEG signals and their inherent variability. By integrating both channel selection and data augmentation, the overall DACS TBTrans model capitalizes on these individual strengths, markedly boosting classification accuracy for nearly all subjects and achieving the best performance for every participant except Subject 5. This combined approach not only elevates individual subject performance but also raises the overall mean classification accuracy, underscoring the synergistic potential of jointly employing these techniques in EEG-based applications.

The data presented in Table 4 highlights the incremental benefits of each component in our methodology. The addition of the channel selection module alone improved the average accuracy by 1.74%, indicating its role in enhancing feature discriminability. The data augmentation component further elevated the average accuracy by 7.33%, demonstrating its effectiveness in enriching the dataset and mitigating overfitting. Most significantly, the combined approach achieved an 8.92% increase in accuracy over the baseline model, affirming the synergistic advantages of integrating both techniques. These results validate our approach’s ability to substantially enhance EEG signal classification accuracy. As shown in the table, the proposed data-augmentation method boosts the baseline accuracy by 7.33%, a marked improvement that confirms its ability to effectively blend the two temporal windows provided by the preprocessing stage and to generate physiologically plausible synthetic EEG signals. Compared with the 1.74% gain achieved by channel selection alone, this enhancement underscores the greater importance of data augmentation for improving classification accuracy.

**Table 4:** Ablation study of proposed method on BCI competition IV 2a dataset.

Method	Sub1	Sub2	Sub3	Sub4	Sub5	Sub6	Sub7	Sub8	Sub9	*Avg*
TBTrans	88.19	59.37	90.97	70.13	62.15	62.50	93.75	90.97	82.98	77.89
CS-TBTrans	90.28	69.44	88.19	79.86	64.93	64.58	90.63	88.19	80.56	79.63
DA-TBTrans	90.97	74.65	93.06	86.46	**84.03**	61.81	96.53	92.01	87.50	85.22
DACS-TBTrans	**91.67**	**74.65**	**94.10**	**88.54**	80.90	**70.83**	**97.57**	**93.06**	**89.93**	**86.81**

Bold values indicate the best performance achieved for that metric.

To validate the effectiveness of the proposed Data Augmentation and Channel Selection (DACS) framework, we evaluated it on three baseline architectures—DeepConvNet, EEGNet, and FBCNet—and compared it with our TBTrans model. All experiments were conducted on the BCI Competition IV 2a dataset without using temporally overlapped windows for data expansion. As reported in Table 5, integrating DACS into each baseline consistently improved classification accuracy, except for FBCNet, where the gain was marginal—likely due to divergent feature-extraction strategies. DeepConvNet, EEGNet, and TBTrans all exhibited improvements exceeding 1%, with our backbone TBTrans achieving a 3.85% boost. These results demonstrate that DACS is readily transferable to alternative baseline models.

**Table 5:** Comparison of baseline models using DACS on the BCI competition IV 2a dataset.

Method	Without DACS	With DACS
	*Avg*	
DeepConvNet (Schirrmeister et al., 2017)	61.96	63.00
EEGNet (Lawhern et al., 2018)	66.71	68.01
FBCNet (Mane et al., 2020)	73.38	74.36
TBTrans	77.89	81.74

We conducted systematic experiments on window length using the BCI Competition IV-2a dataset and evaluated the proposed model under different temporal windows; the results are summarized in Table 6. It can be seen that the model achieves the highest classification accuracy with a 4-s window. Table 7 further reports the parameter count and inference latency, showing that the proposed model is comparable to classic deep-learning architectures such as DeepConvNet in terms of model size, and that both the number of parameters and the latency remain within acceptable limits.

**Table 6:** The impact of time length on accuracy.

Length	*Acc*
2	77.54
3	85.26
4	**86.81**

Bold values indicate the best performance achieved for that metric.

**Table 7:** Comparison of parameter quantity and inference delay.

Method	Parameter (m)	Delay (ms)
DeepConvNet (Schirrmeister et al., 2017)	0.246	24.89
EEGNet (Lawhern et al., 2018)	0.031	3.87
FBCNet (Mane et al., 2020)	0.009	1.23
DACS-TBTrans	0.344	34.17

**Figure 11:**
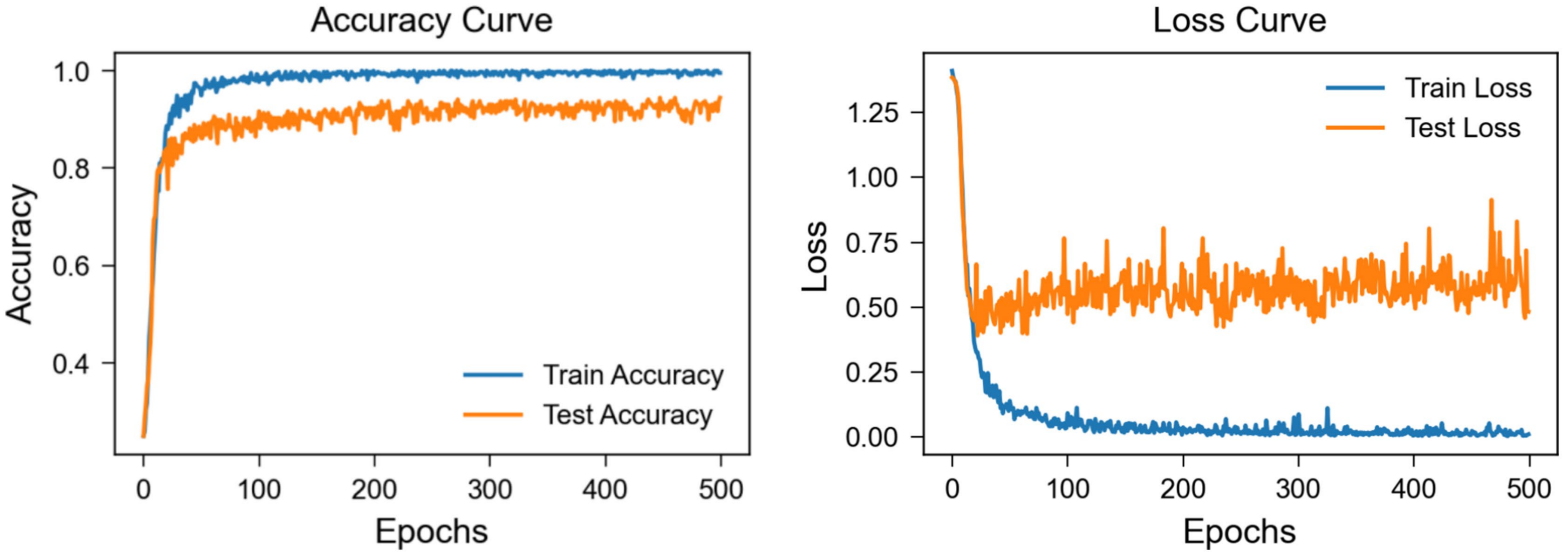
Learning curve of subject 3 on BCI competition IV 2a dataset.

The learning curve graph from Figure 11, based on Subject 3 of the BCI Competition IV 2a dataset, reveals discrepancies between training and testing performance metrics. The training accuracy quickly approaches 100%, while the testing accuracy stabilizes at around 88%, suggesting the potential presence of overfitting. Additionally, the training loss decreases to near zero, yet the testing loss exhibits significant fluctuations and remains relatively high, further indicating a generalization gap. These results underscore the necessity for strategies to mitigate overfitting. In subsequent work, our approach will further refine the model to enhance its robustness and generalization capabilities on unseen data.

**Figure 12:**
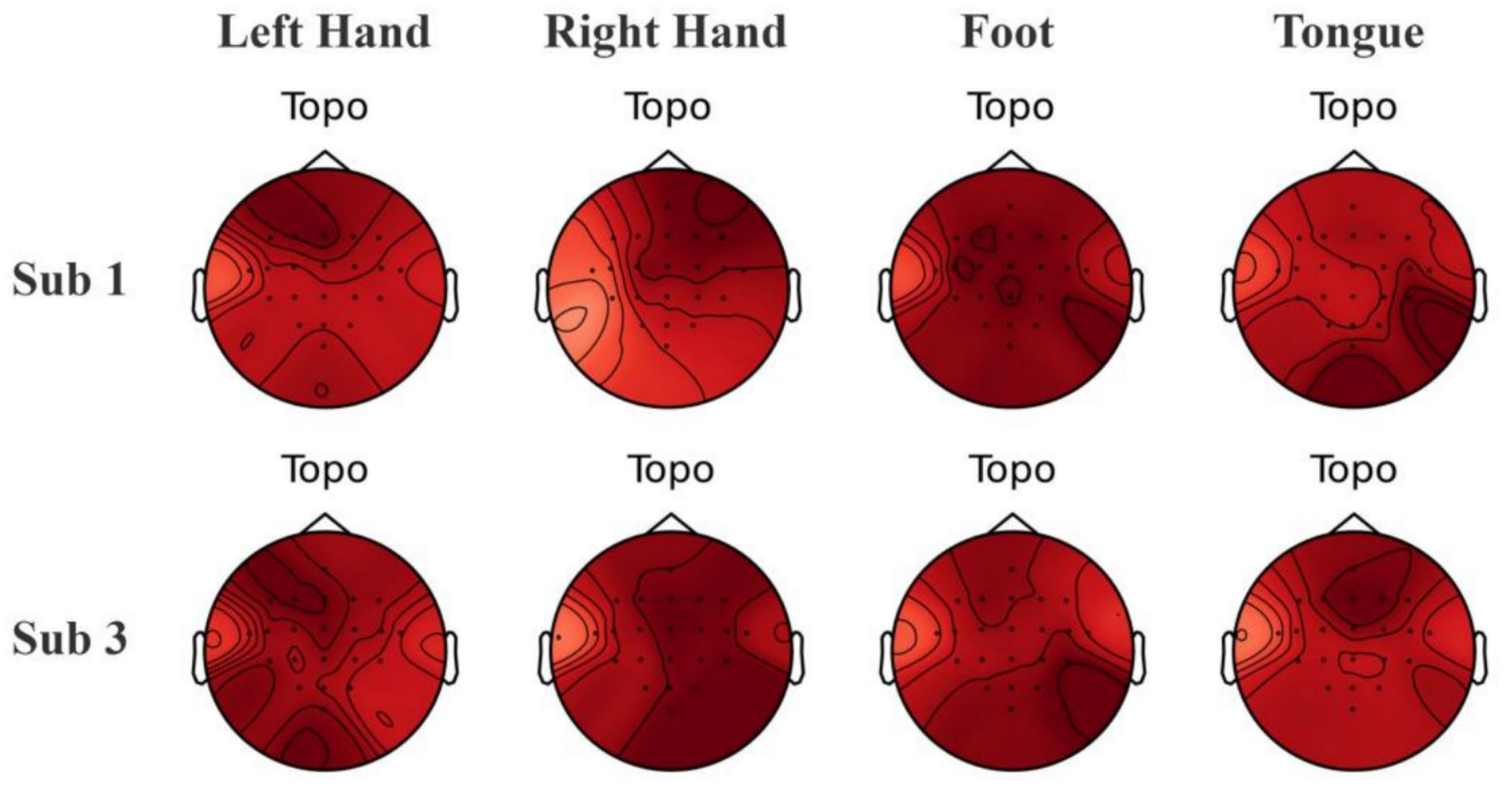
Visualization of brain topography maps of some subjects on the BCI Competition IV 2a dataset.

To explore the interpretability of the features captured by the model, we employed Grad-CAM (Selvaraju et al., 2017) to calculate the contribution of EEG electrode channels to the classification of a particular category and visualized the results. By using backpropagation to only propagate the gradients of the target category, we obtained spatial weights by averaging across the channel dimension. These weights were then combined with the corresponding feature maps through weighted summation and the ReLU function was applied to retain only the positive contributions, ultimately upsampling to produce heatmaps. Grad-CAM heatmaps reveal activations over the C3/C4 region that are consistent with task-related lateralization (Figure 12), aligning with the neurophysiological prior of contralateral motor-cortex engagement and thus enhancing model interpretability. From Figure 12, it can be observed that there is some overlap in the heatmap regions between Subjects 1 and 3 for the same category, which aligns with the similarities expected from neurophysiology. However, the regions of interest for each subject are relatively independent, showing distinct differences.

### 4.3 Parameter experiment

The channel selection method requires optimization of the hyperparameter controlling channel count. Given the comprehensively ranked channel sequence [2, 14, 3, 18, 11, 17, 16, 8, 13, 1, 10, 9, 4, 19, 5, 15, 12, 20, 0, 7, 21, 6], classification experiments were conducted on the baseline model (without data augmentation) by incrementally selecting increasing channel subsets: from the top 1 channel to all 22 channels.

**Figure 13:**
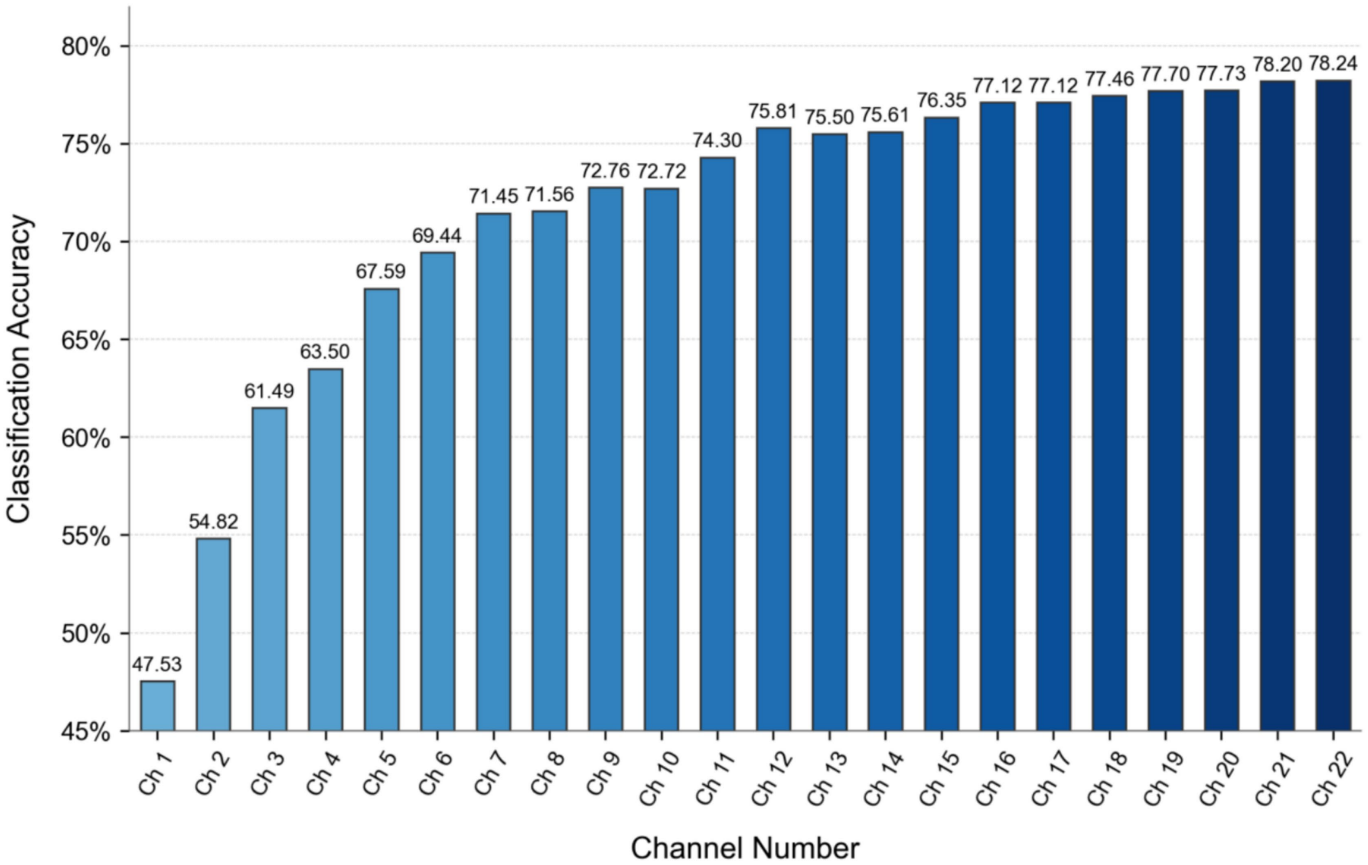
Channel selection hyperparameter optimization results.

As shown in Figure 13, classification accuracy generally improves with increasing channel count until the top 12 channels. Beyond this point, accuracy temporarily decreases when selecting the top 13 and 14 channels, indicating channel redundancy in the middle section of the sequence. Referencing the conventional 16-channel configuration from Common Spatial Pattern (CSP) spatial filtering, we set the channel selection hyperparameter *n* = 16. This configuration combines the top 12 channels with the last four channels of the sequence (indices 0, 7, 21, 6), deliberately bypassing intermediate channels to avoid redundancy.

## 5 Discussion

The DACS-TBTrans method has shown promising results on both the BCI Competition IV 2a and Physionet MI datasets, indicating its potential for EEG-based classification tasks. However, the persistence of significant feature overlap in certain subject-class combinations underscores the need for further refinement in feature extraction methodologies to enhance discriminative capabilities.

**Figure 14:**
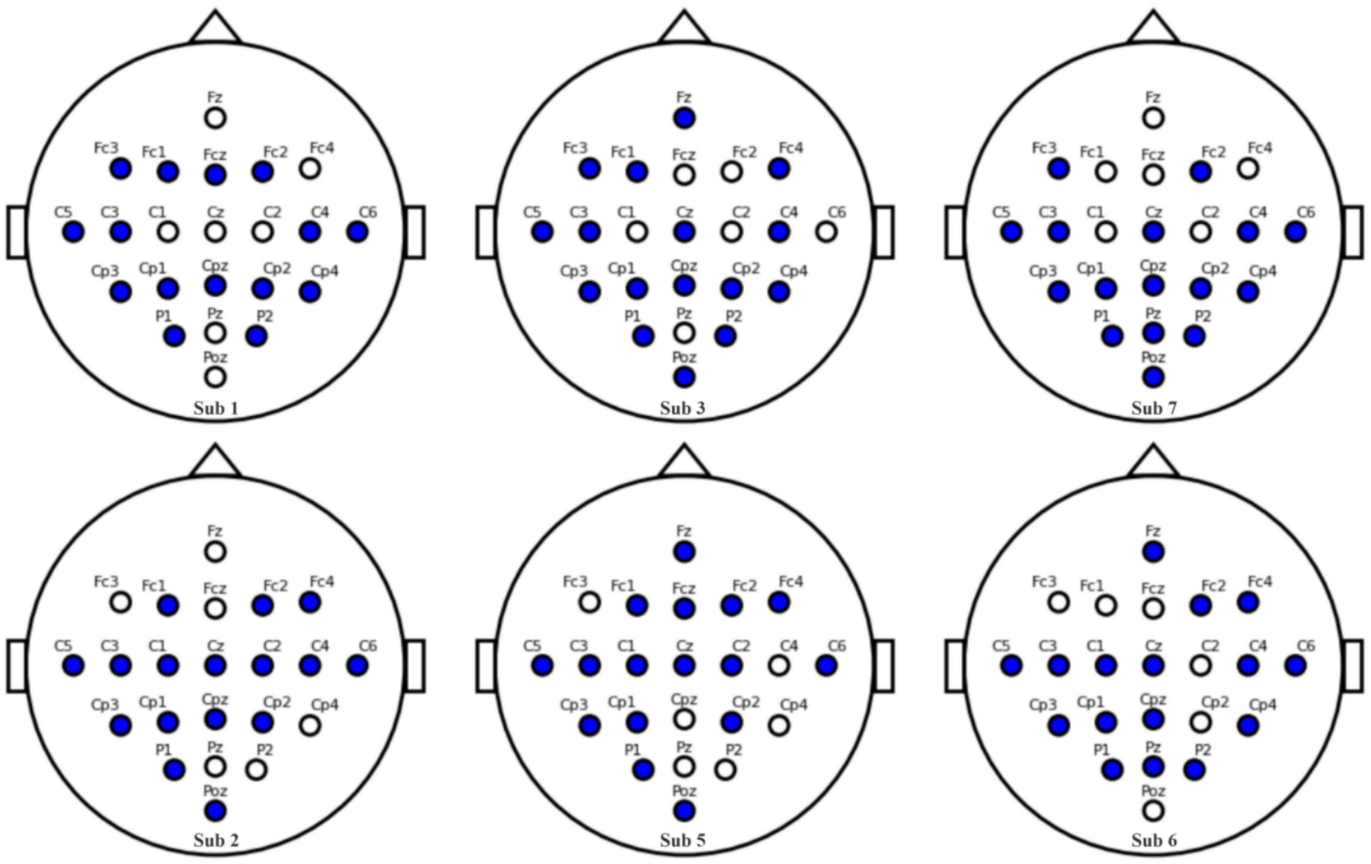
Channel symmetry comparison: Subjects 1, 3, 7 vs. Subjects 2, 5, 6.

Our channel selection experiments, conducted systematically across subjects in the BCI Competition IV 2a dataset, revealed an intriguing correlation between the 12 + 4 channel configuration and classification accuracy. Specifically, an increase in accuracy was observed with greater spatial symmetry of the selected channels. As depicted in Figure 14, we introduce the Channel Symmetry Index (CSI), which quantifies the number of supplementary channels needed for perfect bilateral symmetry per electrode row. Notably, subjects who achieved classification accuracies above 90% (e.g., S1, S3, S7) demonstrated superior channel symmetry with CSI values of 1–2, whereas subjects with lower accuracies (below 70%, such as S2, S5, S6) exhibited higher asymmetry with CSI values of 3 or more. These findings suggest that optimizing channel symmetry could be a valuable strategy for improving EEG signal classification, warranting further investigation into the role of spatial configuration in enhancing EEG-based brain–computer interfaces. Subjects (<70%, S2/S5/S6) showed higher asymmetry (CSI ≥ 3).

**Figure 15:**
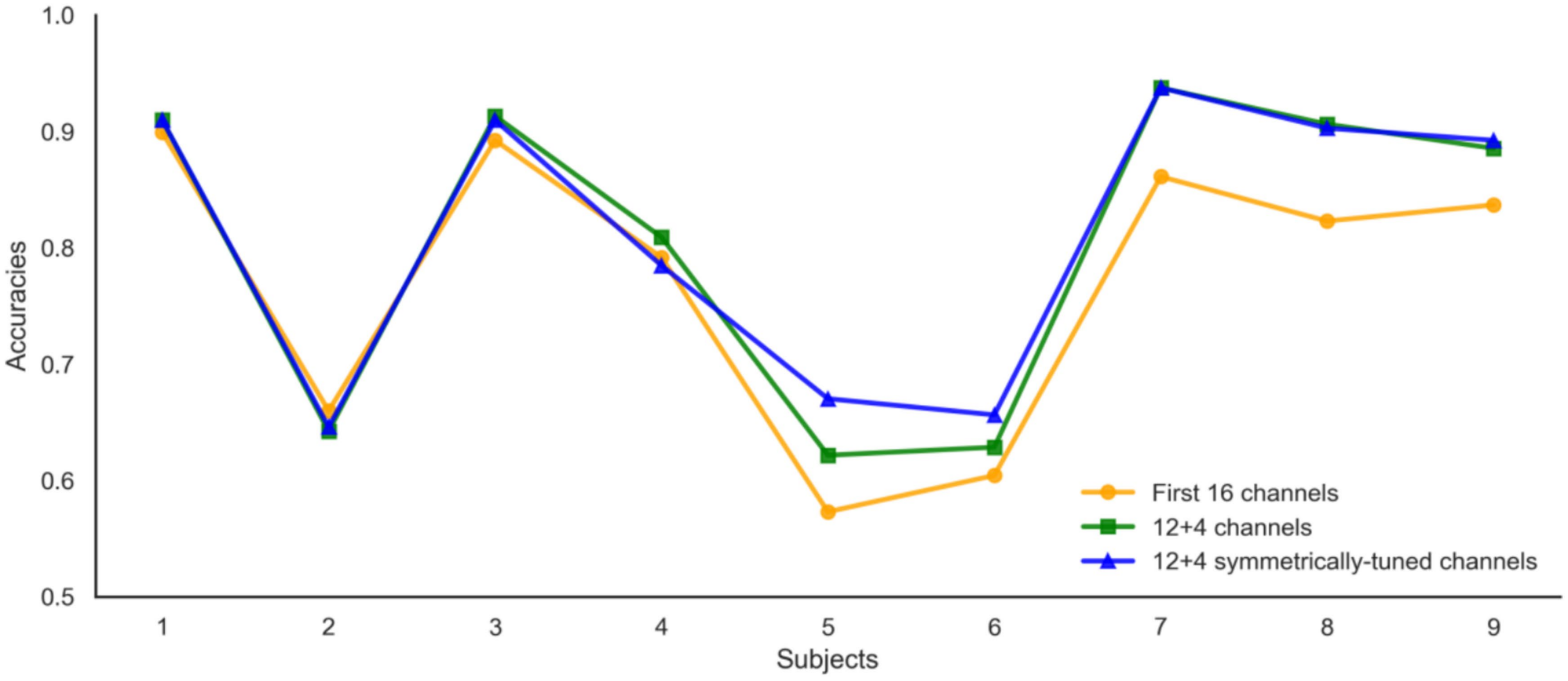
Channel selection results with strategic supplementation.

Figure 15 illustrates the impact of channel configuration on classification accuracy across different subjects. The graph compares the performance of three different channel selection strategies: using the first 16 channels, a 12 + 4 channel configuration, and a 12 + 4 configuration with symmetrically-tuned channels. It is evident that the 12 + 4 symmetrically-tuned channels consistently outperform the other two strategies, particularly for Subjects 5 and 6, where accuracy saw a significant boost. This suggests that enhancing channel symmetry through strategic supplementation of 1–3 channels can substantially improve classification performance. The findings underscore the importance of considering the spatial arrangement of channels in EEG-based classification systems. Future research will focus on further investigating the relationship between channel symmetry and classification performance to optimize EEG signal interpretation and enhance the reliability of brain–computer interfaces.

## 6 Conclusion

This study proposes a motor imagery EEG processing framework tailored for small datasets. By leveraging frequency-domain information—including power spectral density, wavelet packet decomposition/reconstruction, and wavelet packet energy entropy—we generate synthetic EEG data that closely mimics real recordings. Concurrently, channel redundancy is reduced through strategic electrode pruning. Our architecture employs multi-scale convolutional layers for localized feature extraction at varying resolutions, coupled with Transformer networks to capture global temporal dependencies. Evaluated on the BCI Competition IV 2a and PhysioNet MI datasets, the proposed method achieved mean accuracies of 86.81 and 86.64%, respectively. Notably, it outperformed the full-channel baseline (*p* < 0.01, paired *t*-test) while discarding 27% of sensors, and matched or surpassed state-of-the-art benchmarks. By jointly augmenting data and pruning channels, this framework delivers clinically viable, computationally efficient MI decoding with fewer electrodes—eliminating need for additional recordings—thus paving the way for practical low-density BCI systems in real-world applications.

## Author’s note

We will validate the proposed framework on additional public MI datasets (e.g., GigaDB and OpenBMI) to examine cross-dataset generalizability.

## Data Availability

The original contributions presented in the study are included in the article/supplementary material, further inquiries can be directed to the corresponding author.
